# Women’s empowerment and nutritional status of children in the Gambia: further analysis of the 2020 Gambia demographic and health survey

**DOI:** 10.1186/s12889-023-15494-1

**Published:** 2023-03-29

**Authors:** Jainaba Sey-Sawo, Francis Sarr, Haddy Tunkara Bah, Thomas Senghore

**Affiliations:** Department of Nursing and Reproductive Health, University of The Gambia, Serrekunda, Gambia

**Keywords:** Child Health and Development, Gender, Nutrition, Women Empowerment, Stunting, Underweight

## Abstract

Empowering women and the promotion of children’s health are key components of the Sustainable Development Goals targeted for achievement by 2030. The survival of young children, which depends on their nutrition, is influenced by an interaction of factors at the household level. This study aims to investigate the association between women’s empowerment and undernutrition among children under age 5 using The Gambia Demographic Health Survey (GDHS) 2019–20.

Children’s undernutrition was measured with two indicators: stunting and underweight. The women’s empowerment indicators were educational status, employment, decision making, age at first sex and age at first birth, and acceptance of wife beating. StataSE software Version 17 was used for data analysis. Analyses were cluster-adjusted, sample-weighted, with confounding/moderating variables. Descriptive statistics and cross-tabulations were computed for all variables. Bivariate and multivariate analysis of the outcomes and women’s empowerment were conducted.

The prevalence of stunting and underweight among the children under age 5 was 17% and 12%, respectively. The results of the multiple logistic regression show that women with no education had 51% (OR = 1.51; 95% CI = 1.11–2.07; *p* = 0.009), and 52% (OR = 1.52; 95% CI = 1.06–2.14; *p* = 0.022) greater odds of having children under age 5 who were stunted or underweight compared to those women with primary and higher level of education, respectively. Mothers with a body mass index classified as thin were associated with an increased odds of having stunted (OR = 1.44; 95% CI 1.01–2.05; *p* = 0.033) and underweight (OR = 1.69; 95% CI = 1.58–3.52; *P* < 0.001) children. In addition, women who reported accepting wife beating had 69% (OR = 1.69; 95% CI 1.22–2.35; *p* = 0.002) and 66% (OR = 1.66; 95% CI 1.15–2.40; *p* = 0.006) greater odds of having stunted and underweight children respectively compared to those who did not accept wife beating.

In conclusion, the result of this study shows that women’s empowerment is associated with undernutrition among children under age 5 in The Gambia. This is suggesting that implementing policies and interventions that increase the empowerment of women will contribute to the improvement of child nutrition in the country.

## Introduction

The world Health organization estimated in 2020 that worldwide, 149 million children under 5 age were stunted (too short for age), and 45 million were wasted (too thin for height) [1]. This estimate continued to mention that about 45% of deaths among children under 5 years of age were associated with undernutrition and most of these deaths occured in low- and middle-income countries. In 2018, 17.1% (29 million) of children under five age in the African Region were underweight [2]. Despite the data showing that the prevalence of stunting among children under 5 age decreased in the African Region within the 17 years period of the report (2000 and 2017), but the number of affected children rose from 50.6 to 58.7 million due to population growth [3]. The same report indicated that the prevalence of wasting in the African region in 2017 was 13.8 million children, of whom 4 million were severely wasted. Similarly, In The Gambia, 18% of children under 5 age were stunted, 5% were wasted and 12% were underweight [4]. The data is showing that undernutrition still remains a serious global public health problem.

The global momentum on improving `child nutrition has been increasing steadily: the World Health Assembly adopted the 2025 Global Targets for Maternal, Infant and Young Child Nutrition in 2012 and this prompted the 2015 pledge by world leaders to reduce chronic malnutrition in children younger than age 5 by 40% before 2025 [5]. During the first Nutrition for Growth (N4G) Summit which was held in 2013, donors committed US$23 billion to actions to improve nutrition in children [6]. In addition, 2016–2025 was named as the United Nations Decade of Action on Nutrition in the Second International Conference on Nutrition (ICN2) held in 2014 [7]. In 2015, the UN Sustainable Development Goals enshrined the objective of “ending all forms of malnutrition by 2030 [8]. Despite these global efforts, the world is far from achieving the goal of a world without malnutrition. While the 2021 edition of the UNICEF-WHO-World Bank Group Joint Malnutrition Estimates shows that stunting prevalence has been declining since the year 2000, but 149.2 million children under age 5 were stunted in 2020, and 45.4 million suffered from wasting [1].

Empowering women and the promotion of children’s nutrition are among the key components of Sustainable Development Goals (SDGs 2 and 5) targeted for achievement by 2030. However, it has been 25 years now since the adoption of the Beijing Declaration and Platform for equal power and equal rights for women but no country has achieved gender equality, less than 50% of working-age women are in the labour market, and Unpaid domestic and care work falls disproportionately on women [9], In term of power and decision making, women held only 28% of managerial and 22% of cabinet minister positions globally in 2019 (9; 10). In addition, the Global Economic Forum reported that despite the fact that the world is making progress in achieving gender parity in education, but girls still make up a higher percentage of out-of-school children than boys and approximately one quarter of girls in Africa do not attend school [10].

The survival of children depends on their nutrition and is influenced largely by the interaction of factors at the household level [11, 12]. The greater responsibility of caring for children often rests with women who contribute more to this household interaction. This is evident because the empowerment of women is positively correlated with child survival [13]. Empowered women and girls contribute to the health and productivity of their families, communities, and countries, creating a broad effect that benefits everyone [10].

Women’s empowerment is multifaceted and can differ from one cultural context to another. There is limited research on the indicators of women’s empowerment in The Gambian context. Women’s empowerment is described as the process by which a woman achieves agency [14], and where women can access resources that enable them to acquire the capability to articulate preferences and make decisions to meet their own aspirations [15]. These resources include human resource development such as schooling attainment and earned income [15]. Yount et al. found that in Egypt, women’s empowerment includes their ability to influence family decisions, including those reserved for men, freedom of mobility, and attitudes towards gender-based violence, and specifically, violence against wives [16]. The Gambia is a patriarchal society like Egypt where women have fewer opportunities for education, employment, and decision making, as compared to their male counterparts. However, freedom of mobility to public places such as markets may not be considered as part of agency for women’s empowerment in The Gambia because it is part of women’s role to do the household shopping in the markets.

Several studies have explored the relationship between women’s empowerment and children’s nutritional status. These studies show that empowered and educated mothers have a great influence on the reduction of child stunting [17–20]. Another study reported that women’s income is associated with a child’s nutritional status [21]. Similarly, child health was positively associated with many maternal factors such as age, income, household size, access to safe drinking water, and location of the house [22]. Various other studies in Sub-Saharan Africa also showed similar results. For example, women’s empowerment was associated with child health outcomes in Nigeria [17], while mothers’ education was positively associated with a reduction in children’s malnutrition in Tanzania, Zimbabwe, and Malawi [23]. Women who are empowered to make decisions and can contribute to the financial support of the household may have a greater influence on budgets for household food procurement, food allocation in the household and greater agency in how they choose to feed their infants [14].

The Government of the Gambia is committed to the empowerment of women, and has been vigorously pursuing policies that address the health, nutrition, and demographic needs of women. The Government is also committed to improving the nutritional status of children. The Nafa Cash Transfer Program which was launched by the Government of Gambia, in partnership with the World Bank supports and empowers women by encouraging households to choose women as recipients of the cash, for food security, health, education and investments in micro-enterprises. The program also provides specific messaging to children and their families about nutrition, health, and schooling. However, despite these efforts, many challenges remain for women. Malnutrition is also a public health concern. Recent data on The Gambia show that 18% and 12% of children are stunted and underweight, respectively [4]. A comparison of the GDHS 2013 to 2019–20 indicated that under-5 mortality increased from 54 to 56 deaths per 1,000 live births, infant mortality increased from 34 to 42 deaths per 1,000, and neonatal mortality rose from 22 to 29 deaths per 1,000 live births [4, 24]. Women are the primary caretakers of children at the household and community level, and their empowerment affects child wellbeing and survival. Women act as means to improve the nutritional status of children, which is associated with other important developmental outcomes.

Age at first marriage, first sex, first cohabitation, and first birth are enabling resources for women [25]. Child marriage, especially for girls, remains a challenge in The Gambia, where 34.2% of women (age 20–49) are married before age 18 [24]. Early marriage can steal from a girl the opportunity to achieve higher education and skills training, which affect her employability. In The Gambia, some young girls are married at an early age into extended families, where they help their mother-in-laws with the household chores while their husbands travel to other countries for job opportunities. These men frequently travel without the required legal documents, and it can sometimes take up to 5 years or more before they can travel back to The Gambia. This means that early marriage in The Gambia does not always lead to early sexual debut, although the risk is higher. Early sexual debut can result in teenage pregnancy with an increased risk of having an underweight baby and breastfeeding difficulties [4].

Although there are several studies that explored the relationship between women’s empowerment and children’s nutritional status, no study has explored this relationship in the context of The Gambia. The application of findings from other countries to The Gambia may be problematic given that the difference in women’s empowerment levels, which is influenced by cultural and religious factors. Therefore, the study aims to investigate the association between women’s empowerment and malnutrition in children under age 5 using The Gambia Demographic Health Survey (DHS) 2020 data.

### Research question

Our primary research question is: What is the relationship between women’s empowerment and stunting and underweight among children under age 5. We will also describe stunting and underweight in children under age 5 in The Gambia and women’s empowerment in terms of level of education, decision making, employment status, attitude towards wife beating, as well as age at first marriage and birth.

### Conceptual framework

**Fig. 1:**
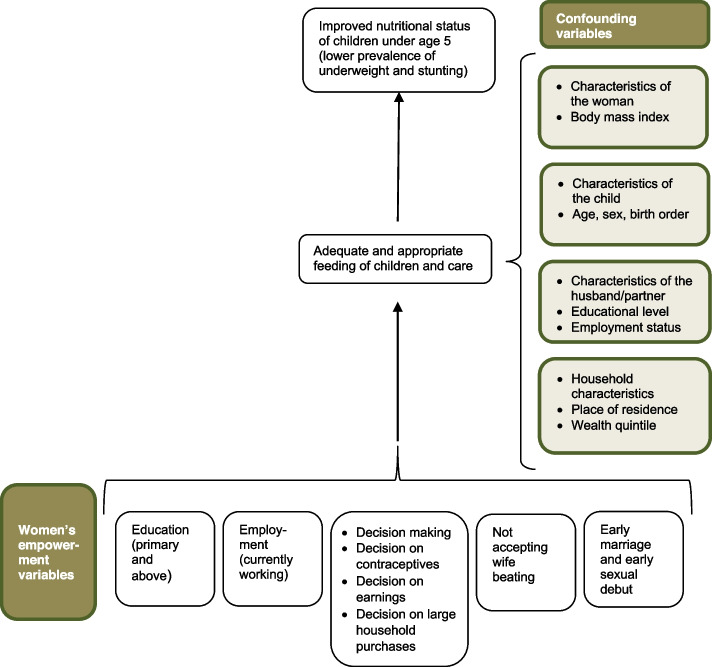
Conceptual model from women’s empowerment to children’s nutritional status

Despite the operational differences in women’s empowerment, several studies have reported the association of women’s empowerment with practices that indirectly affect child nutrition [4, 25, 26]. Our conceptual framework (Fig. 1) which was drawn from these studies and the UNICEF framework for child under nutrition describes how women’s empowerment indicators such as education, employment, decision making, early marriage, and attitudes toward wife beating can influence under-nutrition among children under age 5 in The Gambia. The additional modifying/confounding variables included in the analyses have also been informed by existing literature.

In this study, we expected that households in the rich wealth index quintile would be more likely to provide nutritious food to its members, as compared to those in the poor wealth index quintile [14, 16]. Poverty in the household may also force women to go out of the home to work. Thus, in addition to examining the pathways by which women’s empowerment is associated with child nutritional status, we explored the extent to which household wealth may confound or modify these pathways.

Maternal nutritional status, as measured as body mass index (BMI), was also identified as a confounding variable for the nutritional status of children [25]. The other confounding variables identified in our model include the woman’s husband/partner’s educational level and employment status, and characteristics of the child.

Due to the lack of a standardized measure for women’s empowerment [25, 27] and to avoid masking the differential contributions of specific indicators of empowerment to child nutrition [26], the relationships between the empowerment variables and nutritional status of children under age 5 in the Gambia were analyzed individually, rather than constructing an empowerment index. As empowerment indexes for surveys are being developed, further disaggregated analyses of such indexes are needed to examine the relationship between women’s empowerment indicators and outcomes that include children’s nutrition [28].

### Data and methods

#### Data

The Gambia DHS 2020 was used for this analysis. The methods used in the DHS have been described previously [4]. The survey used a nationally representative sample, and the sampling involved two stages. The country is divided into local government areas. In the first stage, primary sample units, which are called enumeration areas, were selected from local government areas proportional to size. In the second stage, the households were selected. Participants (both men and women) were selected in sampled households for interviews. To answer the research question, we analyzed the GDHS data, which were obtained by interviewing women who were married or living with their partners during the time of the survey, age 15 to 49, and with children under age 5 who had anthropometric measurements. Therefore, the unit of analysis was the child. Any variables that referred to the women were identified as the mother’s characteristics. The KR file includes data for children born to interviewed women in the previous five years (age 0–59 months). This includes data on the children’s demographic characteristics and other health-related characteristics such as stunting and underweight.

### Variables

#### Dependent variables

The children’s nutritional status was measured with two indicators: stunting and underweight. Stunting is measured with height-for-age. Stunting occurs in children whose height-for-age Z-score (HAZ) is less than minus two standard deviations (-2 SD) from the median of the reference population [29]. The HAZ is calculated by subtracting an age and sex-appropriate median value from the standard population and dividing by the SD of the standard population [29]. The children who are stunted are a subset of those with linear growth retardation. Underweight is assessed with the weight-for-age Z-score (WHZ) of the children. The WHZ is a composite index of height-for-age and weight-for-height. Children whose WHZ was below minus two standard deviations (-2 SD) from the median of the reference population were classified as underweight [29].

### Women’s empowerment variables

Women’s empowerment was measured with five indicators: educational level, employment status, decision-making, age at first marriage and sex, and acceptance of wife beating. Women’s participation in decision-making was determined by their responses to questions about who normally decides on large household purchases, contraceptive use, and self-earnings. These decision-making variables were recoded: the responses of the woman only makes decisions and joint decisions with husband were coded as 1, which indicated that the respondent is empowered to make decisions. The responses that indicated only the husband or others made the decisions were coded as zero. This meant that the respondent was not empowered to make decisions. The results from each respondent on the decision-making variables were added after recoding to make a composite variable, which was called empowered to make decision. A total score of 2–3 was coded as 1 for yes, which meant the woman is empowered to make decisions, and a score of 0–1 was coded as zero, which meant not empowered. Acceptance of wife beating was measured by the respondent’s agreement that beating was justified if the wife went out without permission of husband, neglected the children, argued with husband, refused sex with husband, or burned the food. “Yes” responses to these variables were coded as zero, which indicated that the respondent accepted been beaten, while the “No” was coded as 1, which meant the woman did not accept beating under the stated circumstances. All “I don’t know” responses were deleted from the analysis. A variable labeled as “accepts wife beating” was formed by adding scores of each respondent on the acceptance of wife beating variables after recoding them. A total score range from 1–5 was coded as 1 and classified as “respondent does not accept wife beating and is empowered, and a score of zero was coded as zero representing that the respondent accepts wife beating and is not empowered.” The women’s educational level was recoded as primary level and above as educated, and no primary education as non-educated. Women’s employment status was categorized as currently working at the time of interview if the woman reported working 7 days in the past 12 months or not working during the period of the interview.

### Other variables

Other variables included those that could have confounding or moderating effects on the relationship between the empowerment and child’s undernutrition variables. These include the parity of the mother (number of living children), age, sex (male/female), and birth order of the children. The educational level (categorized into two indicators of no education/primary education and above) and employment status of the husbands/partners of the mothers (employed or not employed), nutritional status of the mother (BMI classified as normal weight, thin, overweight, or obese), residential place (rural/urban), and household wealth index (the continuous wealth factor scores were used to calculate tertiles, which were classified as poor, middle, or rich).

### Statistical analysis

The analyses were cluster-adjusted, sample-weighted, and included the important confounding/moderating variables. Weights were applied to assure the sample data were representative of the entire population. Descriptive statistics and cross-tabulations were computed for all variables. Bivariate analysis of the outcome, women’s empowerment, and confounding variables were conducted with the Chi-square test. We used multiple logistic regressions to measure the strength and direction of the relationships between the women’s empowerment, and the confounding and outcome variables. Before conducting the multiple logistic regression analyses, preliminary analyses which tested the assumptions of normality, linearity, multi collinearity, and homogeneity of variance were tested by using the variance inflation factor (VIF) and Pearson correlation tests. The variables with a VIF of 5 or more were not added to multiple logistic regression models [30]. In addition, when two variables had a correlation coefficient of 0.7 and above, only one was selected for the regression model. All variables were added to multiple regressions, except for obesity and normal weight because they have VIF values greater than 5. The 95% confidence intervals and odd ratios were obtained and p-values less than 0.05 were considered as statically significant. Stata SE software version 17 was used to analyze the data.

## Results

### Characteristics of respondents

A total of 7,123 women, age 15 to 49, who were married or living with partners, and had children under age 5, were interviewed and their data analyzed in this study (Table 1). More than half were between age 25 to 34 (54.2%), had primary and above level of education (52%), and were employed (54.2%). Early sex debut at the age between 8 and 16 was 39%, and 51.3% of the women had their first children at the age of 12 to 19. Most of the women had a normal BMI (51.4%). Sixty-nine percent (*n* = 4886) had 1 to 2 living children under age 5. More than half of women (58.1%) reported that they accept wife beating by a husband under certain situations, such as burning the food, going out without permission from husband, neglecting child care, or refusing sex. However, the majority (90%) of the women reported that they were empowered to make decisions about family planning, their earnings, and large household purchases.

**Table 1:** Background characteristics of respondents

Percent distribution of mothers of children under age 5 by selected background characteristics, The Gambia DHS 2019–20		
**Women’s characteristics and empowerment variables**	**Weighted number**	**Weighted percentage**
**Mother’s age in years**		
15–24	1,392	19.5
25–34	3,864	54.2
35–44	1,725	24.2
45–49	142	2.1
Total	7,123	100
**Mother’s educational level**		
None	3,418	48.0
Primary and above	3,705	52.0
Total	7,123	100
**Mother’s employment status**		
None	3,260	45.8
Employed	3,863	54.2
Total	7,123	100
**Mother’s age at first sex in years**		
8–16	2,769	38.9
17–24	4,014	56.3
25–40	341	4.8
Total	7,123	100
**Mother’s age at first birth in years**		
12–19	3,652	51.3
20–27	3,192	44.8
28–48	278	3.9
Total	7,123	100
**Mother has normal weight**		
No	1,530	48.6
Yes	1,619	51.4
Total	3,149	100
**Mother is thin**		
No	2,880	91.4
Yes	269	8.6
Total	3,149	100
**Mother is overweight or obese**		
No	1,888	60
Yes	1,261	40
Total	3,149	100
**Mother accepts wife beating**		
No	2,940	41.9
Yes	4,086	58.1
Total	7,026	100
**Mother is empowered to make decision**		
No	719	10.1
Yes	6,404	89.9
Total	7,123	100
**Characteristics of children**		
**Number of living children**		
1–2	4,886	68.6
3–4	2,237	31.4
Total	7,123	100
**Age of child (in yrs)**		
0–2	4,244	62.5
3 and above	2,548	37.5
Total	6,792	100
**Birth order of child**		
1–5	5,630	79
6–10	1,450	20.4
11 and above	43	0.6
Total	7,123	100
**Sex of child**		
Male	3,676	51.6
Female	3,447	48.4
Total	7,123	100
**Has a stunted child under age 5**		
No	2,717	82.9
Yes	562	17.1
Total	3,279	100
**Has an underweight child under age 5**		
No	2,913	88.2
Yes	390	11.8
Total	3,303	100
**Characteristics of husbands/partners**		
**Husbands’ educational level**		
None	3,557	55.1
Primary and above	2,902	44.9
Total	6,459	100
**Employment status of husband**		
Not employed	257	3.6
Employed	6,789	96.4
Total	7,046	100
**Household characteristics**		
**Household wealth index**		
Poor	1,499	21
Middle	2,387	33.5
Rich	3,237	45.5
Total	7,123	100
**Place of residence**		
Urban	4,569	64.1
Rural	2,554	35.9
Total	7,123	100

Most of the women were located in the urban areas of Gambia (64.1%) and had a wealth index that was classified as rich (45.5%).

The analysis of the data on children showed that majority were between age 0 to 2 (62.5%), and were males (61.3%). With their nutritional status, stunting and underweight were 17% and 12%, respectively.

Most of the husbands/partners of the women were illiterate (55.1%), although almost all were employed (96.4%).

### Bi-variate analysis result

The bi-variate analysis presented in Table 2 shows that the number of living children, household wealth index, thinness of the mother, acceptance of wife beating, birth order of the child, household wealth index and the mother’s husband/partner’s level of education were significantly associated with the prevalence of stunting and underweight among the children under age 5 five. Place of residence and age of the child were significantly associated with stunting, while age of mother at first sex and the overweight or obesity of the mother were significantly associated with the prevalence of underweight among the children under the age of 5. However, there was no significant association between the level of education, employment status, and decision-making among the mothers and any underweight or stunting among the children.

**Table 2:** Factors associated with stunting and underweight among children under age 5, The Gambia DHS, 2019–2020 (*n* = 3279)

	**Stunted child under age 5**				**Underweight child under age 5**			
**Women’s characteristics and empowerment variables**	**No**	**Yes**	**χ2**	**p-value**	**No**	**Yes**	**χ2**	**p-value**
	**%**	**%**			**%**	**%**		
**Age of mother**			0.014	0.994			0.908	0.779
15–24	83.0	17.0			89.2	10.8		
25–34	82.8	17.2			88.1	11.9		
35–49	82.8	17.2			87.7	12.3		
**Level of education of mother**			0.185	0.729				
None	82.6	17.4			87.9	12.1	0.338	0.653
Primary and above	83.1	16.9			88.5	11.5		
**Employment status of mother**			2.723	0.264			0.046	0.893
Not employed	81.7	18.3			88.1	11.9		
Employed	83.8	16.2			88.3	11.7		
**Place of residence**			7.859	0.031*			5.441	0.051
Urban	84.2	15.8			89.1	10.9		
Rural	80.5	19.5			86.5	13.5		
**Number of living children**			10.272	0.020*			15.746	0.006*
1 -2	84.2	15.8			89.7	10.3		
3–4	79.9	20.1			85.1	14.9		
**Age of mother at first sex in years**			4.985	0.268			11.808	0.040*
8–16	81.4	18.6			86	14		
17–24	84.1	15.9			89.7	10.3		
25–40	81	19			89.4	10.6		
**Age of mother at first birth in years**			3.221	0.472			7.852	0.097
12—19	81.8	18.2			86.8	13.2		
20–27	84.1	15.9			89.9	10.1		
28–48	83.1	16.9			87.6	12.4		
**Household wealth**			23.845	0.002*			26.313	0.001*
Poor	85.2	14.8			77.8	22.2		
Middle	86.1	13.9			81.6	18.4		
Rich	90.9	9			85.9	14.1		
**Mother has normal weight**			1.858	0.336			0.02	0.92
No	83.6	16.4			88	12		
Yes	81.7	18.3			87.8	12.2		
**Mother is thin**			6.996	0.021*			28.0359	< 0.001*
No	83.2	16.8			88.8	11.2		
Yes	76.9	23.1			78	22		
**Mother is overweight or obese**			8.6225	0.06			10.416	0.033*
No	81	19			86.3	13.7		
Yes	85.1	14.9			90.2	9.8		
**Mother empowered to make decision**			5.747	0.058			1.698	0.28
No	94.2	5.8			92.5	7.5		
Yes	81.2	18.8			86.2	13.8		
**Mother accepts wife beating**			24.811	0.001*			18.273	0.004*
No	86.5	13.5			90.9	9.1		
Yes	80.1	19.9			86.3	13.7		
**Age of the child in years**			7.855	0.026*			2.007	0.253
0–2	81.5	18.5			88.8	11.2		
3 and above	85.2	14.8			87.2	12.8		
**Birth order of the child**			11.64	0.042*			13.199	0.018*
1–5	83.9	16.1			89.2	10.8		
6–10	78.7	21.3			84.4	15.6		
11 and above	87	13			90.6	9.4		
**Sex of the child**			3.594	0.141			3.08	0.153
Male	81.7	18.3			87.3	12.7		
Female	84.1	15.9			89.2	10.8		
**Husbands’ educational level**			10.049	0.028*			11.497	0.015*
None	81.3	18.7			86.6	13.4		
Primary and above	85.5	14.5			90.4	9.6		
**Husband’s employment status**			1.029	0.472			3.038	0.192
Not employed	86.3	13.7			93.3	6.7		
Employed	82.7	17.3			88	12		

^*^p < 0.05

### Multiple logistic regression result

The results of the multiple logistic regression with adjusted odd ratio in Table 3 show that after controlling for characteristics of the child husband/partner and household, women who reported having no education had 51% (OR = 1.51; 95% CI = 1.11–2.07; *p* = 0.009), and 52% (OR = 1.52; 95% CI = 1.06–2.14; *p* = 0.022) higher odds of having stunted and underweight children under age 5, compared to those with primary and higher level of education, respectively. Mothers with a BMI classified as thin were associated with the increased odds of having stunted (OR = 1.44; 95% CI 1.01–2.05; *p* = 0.033) and underweight (OR = 1.69; 95% CI = 1.58–3.52; *P* < 0.001) children. In addition, women who reported accepting wife beating had 69% (OR = 1.69; 95% CI 1.22–2.35; *p* = 0.002) and 66% (OR = 1.66; 95% CI 1.15–2.40; *p* = 0.006) greater odds of having stunted and underweight children compared to those who did not accept wife beating. Similarly, women from households with a wealth index classified as poor, had 69% (OR = 1.69; 95% CI 1.03–2.80; *p* = 0.038) and 83% % (OR = 1.83; 95% CI 1.18–2.83; *p* = 0.007) greater odds of having underweight and stunted children compared to those from households with middle or rich wealth index. Having a wealth index classified as middle was also significantly associated with greater odds (OR = 1.79; 95% CI 1.13–2.86; *p* = 0.0148) of having stunted children compared to those with a wealth index classified as rich. However, the employment status, age at first sex and birth, and decision-making among the women were not significantly associated with stunting and underweight among their children under age 5.

**Table 3:** Multiple logistic regression results on predictors of stunting and underweight among children under age 5, The Gambia DHS, 2019–2020 (*n* = 3279)

Women’s characteristics and empowerment variables	Stunted child								Underweight child					
	**Odds ratio**	**std. err**	**t**		**P > t**	**95% conf. Interval**			**Odds ratio**	**std. err**	**t**	**P > t**	**95% conf. interval**	
**Age of mother (in years)**														
15–24	0.99	0.36	-0.03		0.975	0.48		2.03	0.79	0.27	-0.67	0.501	0.40	1.56
25–34	1.32	0.325	1.14		0.255	0.82		2.15	1.20	0.33	0.69	0.49	0.71	2.05
**Educational level of mother**														
Primary and above	1.51	0.24	2.61		0.009*		1.11	2.07	1.52	0.27	2.31	0.022*	1.06	2.14
**Residential area of mother**														
Rural	0.89	0.17	-0.61		0.541	0.62		1.33	0.82	0.15	-1.12	0.248	0.58	1.16
**Household wealth index**														
Poor	1.69	0.43	2.09	0.038*		1.03		2.80	1.83	0.41	2.72	0.007*	1.18	2.83
Middle	1.79	0.42	2.48	0.014*		1.13		2.86	1.37	0.29	1.48	0.14	0.90	2.09
**Employment status of mother**														
Not employed	0.79	0.11	1.66		0.097	0.60		1.04	0.96	0.19	-0.22	0.823	0.65	1.40
**Age at first sex of mother in years**														
8–16	0.73	0.30	0.75		0.457	0.33		1.64	1.19	0.53	0.4	0.689	0.50	2.85
17–24	0.76	0.31	-0.69		0.490	0.34		1.68	1.00	0.39	-0.01	0.998	0.47	2.14
**Age at first birth of mother in years**														
12–19	0.88	0.40	0.29		0.773	0.36		2.15	0.64	0.23	1.24	0.215	0.32	1.30
20–27	0.84	0.39	-0.39		0.699	0.33		2.09	0.56	0.20	-1.59	0.113	0.28	1.15
**Mother is thin**														
Yes	1.44	0.26	2.01	0.033*		1.01		2.05	2.36	0.49	4.18	< 0.001*	1.58	3.54
**Mother accepts wife beating**														
Yes	1.69	0.28	3.14		0.002*		1.22	2.35	1.66	0.31	2.76	0.006*	1.15	2.40
**Mother empowered to make decision**														
No	1.15	0.18	0.86		0.391	0.84		1.57	0.98	0.17	0.14	0.89	0.69	1.38
**Number of living children (under age 5)**														
3–4	1.27	0.25	1.19		0.234	0.86		1.88	1.28	0.32	1.00	0.32	0.78	2.09
**Age of child in years**														
3 and above	0.79	0.12	-1.61		0.110	0.59		1.05	1.19	0.17	1.18	0.24	0.89	1.57
**Sex of the child**														
Female	0.76	0.1	-1.97		0.05	0.59		0.99	0.85	0.13	-1.13	0.261	0.63	1.13
**Birth order of child**														
3–4	1.29	0.31	1.05		0.295	0.80		2.07	1.21	0.34	0.68	0.496	0.70	2.10
5–6	0.79	0.50	-0.36		0.718	0.23		2.73	0.96	0.54	-0.07	0.941	0.31	2.92
**Educational status of husband**														
Primary and above	0.83	0.15	-0.05		0.294	0.59		1.17	0.86	0.17	-0.77	0.443	0.59	1.26
**Employment status of husband**														
Not employed	0.95	0.36	0.14		0.886	0.45		2.01	1.58	0.79	0.91	0.362	0.59	4.23

^*^p < 0.05

## Discussion

This study investigated the associations between children’s undernutrition and women’s empowerment indicators using nationally representative data from The Gambia DHS. In multiple logistic regressions that adjust for confounders, we found significant associations between low women’s educational level and acceptance of wife beating with stunting and underweight among the children under age five. We also found significant associations between the women’s low BMI and low household wealth with child stunting and underweight.

Our finding that illiterate mothers had an increased chance of having undernourished children as compared those mothers with primary and higher levels of education is consistent with the literature. A similar finding was reported in a study of women’s empowerment and its association with maternal nutrition and birth weight in Bangladesh [19]. A systematic review conducted in several African countries reported that low maternal education was consistently associated with undernutrition [23, 31]. Similar findings have also been reported in several South Asian countries [32].

Women’s education has implications that go far beyond the classroom. Women’s education translates to improved child health and nutrition through multiple pathways, such as increased autonomy, and enhanced literacy and analytical skills, which can improve the mother’s health and care-giving decision-making, as well as financial decision-making in the household. While significant steps have been taken to improve the empowerment of women in The Gambia through several legislative acts and vigorous efforts to ensure gender parity in primary education, the educational level among the Gambian women continues to lag significantly. A total of 48% of the women in this study were not educated, and this represents a missed opportunity to improve the lives of women and their children [33]. Although The Gambia Education Policy is aligned with the Sustainable Development Goal 4, which focuses on accessible, equitable, and inclusive quality education for all, many female children are excluded from formal education because of family choice of educating males and social norms that drive child marriage [33]. This calls for tailor-made interventions that are gender sensitive.

The acceptance of wife beating among women and greater odds of stunting and wasting among the children remained statistically significant after controlling for several demographic characteristics. Evidence from the 2020 Gambia DHS shows that 51% of women of reproductive age agree that wife battery can be justified [4]. The supportive attitude towards wife beating among women in this study suggests a degree of social acceptance of such practice in The Gambia. The Gambia is a patriarchal society and these attitudes may reflect a cultural norm based on gender inequalities. The perception of most women and girls has been influenced by their lower status in society than that of men and boys on the expectation that they should fulfill certain gender roles [34]. Positive attitudes toward wife beating were associated with intimate partner violence and in the Gambia DHS 2020, 46% of women reported experiencing physical violence at least once since age 15 [4]. A violent family environment can negatively affect women’s physical and mental health, which can adversely affect their caregiving capacities and children’s nutrition [35]. Improving the status of women in Gambia not only reduces the risk for intimate partner violence, but also can have significant influence on child nutrition [36, 37].

Maternal thinness predicted undernutrition among children in our study. This finding aligns with other studies and illustrates how maternal nutritional status during and after pregnancy is important [25, 31, 38]. Failure of the growing fetus to receive adequate nutrients can result in intrauterine growth restriction and low birth weight [31, 37, 38]. In addition, postnatally and in the early childhood years, if suboptimal environmental conditions persist and there are no interventions, women’s continued poor nutritional status can negatively affect infant feeding practices and child growth [39]. The interrelationship between maternal and child nutritional status stresses the value of maternal nutritional status because this can improve both maternal and child health outcomes [40].

We found positive associations between low household wealth index and underweight and stunting among the children. Women from households with a wealth index classified as poor had greater odds of having underweight and stunted children compared to those from households with a middle or rich wealth index. Having a wealth index classified as middle was also significantly associated with having stunted children compared to those with a wealth index classified as rich. Similar findings were reported in several studies conducted in Sub-Saharan Africa [41–43]. The association between a low wealth index and undernutrition in children can be explained by the fact that poor households may not have enough money to provide a balanced diet for the family members. Such families tend to live in poor environmental conditions, have an increased risk to disease exposure, and lack access to basic health care [22, 31,44].

Childhood undernutrition can also worsen the poverty level of a household and that of the country as a whole. According to the findings of one study on the social and economic impact of child undernutrition in the Gambia, families bear 38% of the healthcare costs associated with undernutrition, while the remaining 62% is borne by the health system [45]. Although the families of undernourished children incur a high percentage of the health costs related to undernutrition, the burden of this phenomenon is still an important expenditure in the public sector. Thus, there is a need to include human capital development strategies in all interventions focused on preventing undernutrition in children under age 5 in The Gambia.

### Strength and limitations of the study

This is the first study of the association of women’s empowerment with the nutritional status of children in the Gambia. The study was based on nationally representative data, and the findings can be generalized to guide policy review and interventions on child nutrition in The Gambia. However, our analyses were based on cross-sectional survey data, which limits our ability to test the causal relationship between women’s empowerment and child undernutrition. The infant feeding variables are available in the DHS and their exclusion in this study is a limitation. The feeding variables in this study were excluded because we wanted to maintain our sample size since the indicators are among children age 0 to 2 years or 6 to 23 months. We suggest that future research could conduct a path analyses to examine how each component of women’s empowerment is related to child underweight and stunting to better understand the individual contribution of the women’s empowerment variables.

## Conclusion

The study findings provide evidence of women’s empowerment indicators and their association with underweight and stunting among children under age 5 in The Gambia. The study highlights the importance of maternal nutrition and poverty on child under nutrition. When women are disempowered in society, there are negative health consequences that extend beyond women to children and all of society. Increasing our knowledge of how women’s empowerment affects child nutritional status will hopefully add impetus and insight to public health interventions in the country. Policies and interventions that increase the empowerment of women would contribute to the improvement of child health in the country. Further interventional and longitudinal studies are needed to evaluate the direct impact of women’s empowerment on the nutritional status of children.

## Data Availability

Data is available with the DHS Program, Demography and Health Survey and can be access at https://www.dhsprogram.com/data/available-datasets.cfm

## References

[1] WHO (2021). Malnutrition factsheet. Retrieved at: https://www.who.int/news-room/questions-and answers/item/malnutrition#:~:text=Malnutrition%20affects%20people%20in%20every%20country.%20Around%201.9,million%20are%20stunted%20and%2050%20million%20are%20wasted. Retrieved date- 8th/2/2023

[2] Africa Check (2019). FACTSHEET: Child undernutrition in Africa. Retrieved https://africacheck.org/fact-checks/factsheets/factsheet-child-undernutrition-africa.

[3] UNICEF/ World Health Organization/World Bank. Joint child malnutrition estimates, 2018 edition. http://www.who.int/nutgrowthdb/estimates2017/en/.

[4] Gambia Bureau of Statistics & ICF. The Gambia Demographic and Health Survey 2019–2020 report. Retrieved at: www.gbosdata.org › downloads-file › the-2019–20

[5] de Onis M, Dewey KG, Borghi E, et al. The World Health Organization’s global target for reducing childhood stunting by 2025: rationale and proposed actions. Matern Child Nutr. 2013;9(Suppl 2):6–26. 10.1111/mcn.12075. PMID:24074315;PMCID:PMC6860845. 10.1111/mcn.12075 PMC6860845 24074315

[6] Nutrition for Growth (2013). Nutrition for Growth Summit. https://nutritionforgrowth.org/

[7] United Nations: United Nations Decade of Action on Nutrition.(2014). Retrieved at https://www.un.org/nutrition/about

[8] United Nations. Department of Economic and Social Affairs; Sustainable Development.. https://sdgs.un.org/goals

[9] United Nations (2020). The World’s Women 2020: Trends and Statistics. Retrieved at https://www.un.org/en/desa/world%E2%80%99s-women-2020

[10] World Economic Forum (2018). Gender gap report 2018. Retrieved https://www.weforum.org/reports/the-global-gender-gap-report-2018. 22.

[11] Saaka M (2020). Women’s Decision-Making Autonomy and Its Relationship with Child Feeding Practices and Postnatal Growth. Journal of Nutrition Science.

[12] Tome, J., M.N.N. Mbuya, R.R. Makasi, R. Ntozini, A.J. Prendergast, K.L. Dickin, G.H. Pelto, M.A. et al. “Maternal Caregiving Capabilities are Associated with Child Linear Growth in Rural Zimbabwe.” Maternal & Child Nutrition, 2021, 17 (2): e13122. 10.1111/mcn.13122 10.1111/mcn.13122 PMC7988870 33350100

[13] Shafiq A, Hussain A, Asif M, Hwang J, Jameel A, Kanwel S (2019). The Effect of ‘Women’s Empowerment’ on Child Nutritional Status in Pakistan. International Journal of Environmental Research in Public Health.

[14] Carlson G.J., K. Kordas, and L.E. Murray-Kolb. “Associations between Women’s Autonomy and Child Nutritional Status: A Review of the Literature.” Maternal Child Nutrition, 2015, 11 (4): 452–82. https://onlinelibrary.wiley.com/doi/epdf/10.1111/mcn.12113 10.1111/mcn.12113 PMC6860340 24521434

[15] Kabeer N (1999). Resources, Agency, Achievements: Reflections on the Measurement of Women’s Empowerment. Dev Chang.

[16] Yount K M, KE VanderEnde, S Dodell, YF Cheong. “Measurement of Women’sAgency in Egypt: A National Validation Study.” Social Indicators Research, 2016, 128 (3): 1171–1192. 10.1007/s11205-015-1074-7. 10.1007/s11205-015-1074-7 PMC5010232 27597801

[17] Ibrahim, A., S. Tripathi, and A. Kumar. “The Effect of Women’s Empowerment on Child Health Status: Study on Two Developing Nations.” International Journal of Scientific and Research Publications, 2015, 5 (4):1–8. https://www.ijsrp.org/research-paper-0415/ijsrp-p4005.pdf.

[18] Irena AH, Mwambazi M, Mulenga V (2011). Diarrhea is a Major Killer of Children with Severe Acute Malnutrition Admitted to Inpatient Set-Up in Lusaka, Zambia. Nutr J.

[19] Siddhanta, A., and A. Chattopadhyay.“Role of Women’s Empowerment in Determining Child Stunting in Eastern India and Bangladesh.” Social Science Spectrum 2017, 3 (1): 38–51. http://socialspectrum.in/index.php/sp/article/view/93.

[20] Zereyesus YA, Amanor-Boadu V, Ross KL, Shanoyan A (2017). Does Women’s Empowerment in Agriculture Matter for Children’s Health Status? Insights from Northern Ghana. Soc Indic Res.

[21] Alderman H, Haddad L, Headey DD, Smith L (2014). Association between Economic Growth and Early Childhood Nutrition. Lancet Global Health.

[22] Poda GG, Hsu CY, Chao JC. Factors associated with malnutrition among children <5 years old in Burkina Faso: evidence from the Demographic and Health Surveys IV 2010. Int J Qual Health Care. 2017;29(7):901–8. 10.1093/intqhc/mzx129. 10.1093/intqhc/mzx129 29045661

[23] Makoka, D. 2013. The Impact of Maternal Education on Child Nutrition: Evidence from Malawi, Tanzania, and Zimbabwe. DHS Working Papers No. 84. Calverton, Maryland, USA: ICF International. https://dhsprogram.com/publications/publication-wp84-working-papers.cfm.

[24] Gambia Bureau of Statistics & ICF. The Gambia Demographic and Health Survey 2013 report. https://dhsprogram.com/pubs/pdf/FR289/FR289.pdf

[25] Jones R, Haardörfer R, Ramakrishnan U, Yount KM, Miedema S, Girard AW. Women's empowerment and child nutrition: The role of intrinsic agency. SSM Popul Health. 2019;9:100475. 10.1016/j.ssmph.2019.100475. 10.1016/j.ssmph.2019.100475 PMC6978483 31993480

[26] Onah MN (2021). Women’s Empowerment and Child Nutrition in South-Central Asia; How Important is Socioeconomic Status?. SSM - Population Health.

[27] Santoso MV, Kerr RB, Hoddinott J, Garigipati P, Olmos S, Young SL (2019). Role of Women’s Empowerment in Child Nutrition Outcomes: A Systematic Review. Adv Nutr.

[28] Alkire, S., R. Meinzen-Dick, A. Peterman, A.R. Quisumbing, G. Seymour, and A. Vaz. 2013. The Women’s Empowerment in Agriculture Index. Oxford: Oxford Poverty & Human DevelopmentInitiative. https://www.ophi.org.uk/wp-content/uploads/ophi-wp-58.pdf.

[29] World Health Organization (WHO) Multicentre Growth Reference Study Group. “WHO Child Growth Standards Based on Length/Height, Weight and Age.” Acta Paediatrica, 2006, 95 (Suppl 405): 76–85. 10.1111/j.1651-2227.2006.tb02378.x

[30] Sohil F, Umair Sohali M, Shabbir J. An introduction to statistical learning with applications in R. Statistical Theory and Related Fields, Taylor & Francis Journals. 2022;6(1):87. 10.1080/24754269.2021.1980261.

[31] Akombi BJ, Agho KE, Hall JJ, Wali N, Renzaho AMN, Merom D (2017). Stunting, Wasting and Underweight in Sub-Saharan Africa: A Systematic Review. International Journal of Environmental Research in Public Health.

[32] Wali N., Agbo K.E., and Renzaho A.M. “Factors Associated with Stunting among Children under 5 Years in Five South Asian Countries (2014–2018): Analysis of Demographic Health Survey.”Nutrients, 2020, 12 (12): 3875. 10.3390/nu12123875 10.3390/nu12123875 PMC7767090 33352949

[33] Ministry of Finance and Economic Affairs. 2022. “Empowering the Gambian Woman to Realize Her Full Potential.” https://mofea.gm/empowering-woman.

[34] UNICEF 2022. “Attitude and Social Norms on Violence”. https://data.unicef.org/topic/child-protection/violence/attitudes-and-social-norms-on-violence/.

[35] U.S Department of Health and Human Services 2020. “Effects of Violence against Women.” https://www.womenshealth.gov/relationships-and-safety/effects-violence-against-women.

[36] Alhusen JL, Ray E, Sharps P, Bullock L. Intimate partner violence during pregnancy: maternal and neonatal outcomes. J Womens Health (Larchmt). 2015 Jan;24(1):100–6. doi: 10.1089/jwh.2014.4872. Epub 2014 Sep 29. PMID: 25265285; PMCID: PMC4361157. 10.1089/jwh.2014.4872 PMC4361157 25265285

[37] World Health Organization (WHO). 2010. Preventing Intimate Partner and Sexual Violenceagainst Women: Taking Action and Generating Evidence. Geneva: World Health Organization. https://apps.who.int/iris/handle/10665/44350.

[38] Young MF, Nguyen PH, Casanova IG, Addo OY, Tran LM, Nguyen S, Martorell R, Ramakrishnan U (2018). Role of Maternal Preconception Nutrition on Offspring Growth and Risk of Stunting Across The First 1000 Days n Vietnam: A Prospective Cohort Study. PLoS ONE.

[39] Victora CG, Christian P, Vidaletti LP, Gatica-Domínguez G, Menon P, Black RE (2021). Revisiting Maternal and Child Undernutrition in Low-Income and Middle-Income Countries: Variable Progress Towards an Unfinished Agenda. The Lancet.

[40] Negash, C., S.J.Whiting, C.J. Henry, T. Belachew, and T.G. Hailemaria. 2015. “Association between Maternal and Child Nutritional Status in Hula, Rural Southern Ethiopia: A Cross Sectional Study. PLoS One, 2015, 10(11): e0142301. 10.1371/journal.pone.0142301 10.1371/journal.pone.0142301 PMC4654505 26588687

[41] Adekanmbi VT, Kayode GA, Uthman OA (2013). Individual and Contextual Factors Associated with Childhood Stunting in Nigeria: A Multilevel Analysis. Matern Child Nutr.

[42] Aheto JMK, Keegan TJ, Taylor BM, Diggle PJ (2015). Childhood Malnutrition and Its Determinants among Under-Five Children in Ghana. Matern Child Nutr.

[43] Chirande L, Charwe D, Mbwana H, Victor R, Kimboka S, Issaka AI, Baines SK, Dibley M, Agho KE (2015). Determinants of Stunting and Severe Stunting among Under-Fives in Tanzania: Evidence from The 2010 Cross-Sectional Household Survey. BMC Pediatr.

[44] Alderman H, Headey D (2018). The Timing of Growth Faltering has Important Implications for Observational Analyses of the Underlying Determinants of Nutrition Outcomes. PLoS ONE.

[45] World Food Programme. 2020.The Cost of Hunger: The Social and Economic Impact of Child Undernutrition in The Gambia. https://docs.wfp.org/api/documents/WFP-0000119677/download/.-z.

